# Hyaluronic Acid Influence on Normal and Osteoarthritic Tissue-Engineered Cartilage

**DOI:** 10.3390/ijms19051519

**Published:** 2018-05-19

**Authors:** Shabnam Hemmati-Sadeghi, Jochen Ringe, Tilo Dehne, Rainer Haag, Michael Sittinger

**Affiliations:** 1 Charité—Universitätsmedizin Berlin, Corporate Member of Freie Universität Berlin, Humboldt-Universität zu Berlin and Berlin Institute of Health, Berlin-Brandenburg School for Regenerative Therapies, 10117 Berlin, Germany; Shabnam.hemmati-sadeghi@charite.de; 2 Institut für Chemie und Biochemie, Freie Universität Berlin, 14195 Berlin, Germany; haag@zedat.fu-berlin.de; 3 Charité—Universitätsmedizin Berlin, Corporate Member of Freie Universität Berlin, Humboldt-Universität zu Berlin and Berlin Institute of Health, Tissue Engineering Laboratory, Berlin-Brandenburg Center for Regenerative Therapies & Department of Rheumatology and Clinical Immunology, 10117 Berlin, Germany; jochen.ringe@charite.de (J.R.); tilo.dehne@charite.de (T.D.)

**Keywords:** hyaluronic acid, osteoarthritis, in vitro model, microarray

## Abstract

The aim of this study is to identify gene expression profiles associated with hyaluronic acid (HA) treatment of normal and osteoarthritis (OA)-like tissue-engineered cartilage. 3D cartilage micromasses were treated with tumour necrosis factor-α (TNF-α) (OA-inducer) and/or HA for 7 days. Viability was examined by PI/FDA staining. To document extracellular matrix (ECM) formation, glycosaminoglycans (GAG) were stained with Safranin-O and cartilage-specific type II collagen was detected immunohistochemically. Genome-wide gene expression was determined using microarray analysis. Normal and OA-like micromasses remained vital and showed a spherical morphology and homogenous cell distribution regardless of the treatment. There was no distinct difference in immunolabeling for type II collagen. Safranin-O staining demonstrated a typical depletion of GAG in TNF-α-treated micromasses (−73%), although the extent was limited in the presence of HA (−39%). The microarray data showed that HA can influence the cartilage metabolism via upregulation of *TIMP3* in OA-like condition. The upregulation of *VEGFA* and *ANKRD37* genes implies a supportive role of HA in cartilage maturation and survival. The results of this study validate the feasibility of the in vitro OA model for the investigation of HA. On the cellular level, no inhibiting or activating effect of HA was shown. Microarray data demonstrated a minor impact of HA on gene expression level.

## 1. Introduction

Hyaluronic acid (HA) is a main component of articular cartilage, which provides the backbone of large proteoglycan complexes. HA binds to a cluster of differentiation 44 (CD44) receptors and this binding inhibits interleukin (IL)1β expression and leads to a decline in matrix metalloproteinase (MMP) 1, 2, 3, 9 and 13 production [1]. Moreover, HA, lubricin and phospholipid species endow synovial fluid with its viscoelastic properties and contribute independently or together to lubrication of the articular surfaces [2,3]. As osteoarthritis (OA) progresses, natural concentration of HA and lubricin as well as the molecular weight distribution of HA shift toward the lower ranges, which deteriorates the mechanical/viscoelastic properties of the synovial fluid [3]. Intra-articular HA administration has aimed to restore these properties [4], although there is controversy over its underlying attributes. Apart from shock absorption and joint lubrication, some therapeutic mechanisms of HA in the OA joint are also proposed such as protective effects on cartilage, enhancement of proteoglycan synthesis and reduction of inflammation [5]. The proposed mechanisms and their controversial discussion encouraged us to investigate the manifold effects of HA on normal and OA-like tissue-engineered cartilage on the cellular and molecular level. Our general aim is to create a better understanding of how intra-articular HA treatment could provide therapeutic effects.

In order to address this question we used an established in vitro OA model which offers a high-throughput analysis of potential active substances in a reproducible and very well characterized approach under standardized conditions [6,7]. In 2014, Schlichting et al. [7] overcame the low availability of human primary tissue and disadvantages of animal models by using cells from porcine cartilage sources. They thus developed an easy to manage OA model by introducing tumour necrosis factor-α (TNF-α) into a 3D-micromass culture, which has already been shown to mimic essential aspects of human chondrocyte and native cartilage biology, pathophysiology and differentiation. TNF-α addition established a degradative environment in line with the generation of macroscopic changes such as extensive proteoglycan loss as an implicit feature of human OA. Furthermore, gene expression profiling of porcine tissue-engineered cartilage micromasses revealed human OA reaction pattern such as extensive ECM loss (*collagen type 2* (*COL2A1*), *collagen type 9* (*COL9A1*), *cartilage oligomeric compound* (*COMP*), *aggrecan* (*ACAN*)), cell death, formation of an inflammatory environment through the induction of genes coding for chemokines (*interleukin 8* (*IL8*), *C-C motif chemokine 2* (*CCL2*)) and OA-relevant enzymes (*matrix metalloproteinase1*, *13* (*MMP1, 13*)) and the modulation of genes involved in skeletal development [7].

To study the effects of HA on cartilage formation and maintenance, 3D chondrocyte micromasses were cultured for 14 days to form extracellular matrix (ECM) and were stimulated for further 7 days with HA under normal and OA-like conditions (by adding TNF-α). Parameters such as cell survival, ECM formation, or changes in gene expression profiles were investigated to evaluate the physiologic action of HA on the cellular and molecular level.

## 2. Results

### 2.1. Effect of Hyaluronic Acid on Chondrocyte Viability and Extracellular Matrix Formation

**Figure 1:**
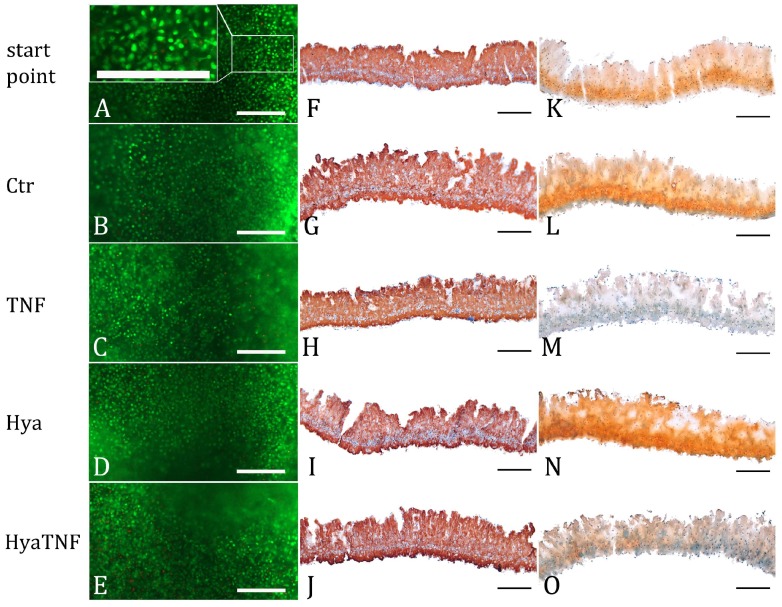
Chondrocyte Viability and Cartilage Quality. (**A**–**E**) Live-dead staining of micromasses of all 5 experimental groups. Living cells were stained green using FDA; dead cells were stained red using PI. (**F**–**J**) Immunohistochemistry demonstrated the presence of cartilage-specific collagen type II (red) as a component of the formed matrix. (**K**–**O**) Safranin-O staining documented the proteoglycan content orange; scale bar represents 200 μm.

To examine the HA effect on normal and OA-like tissue-engineered cartilage on cellular level we obtained fluorescent images from the live/dead assay which demonstrated that all tissue-engineered chondrocyte micromasses in this study remained vital. (green; Figure 1A–E). Furthermore, the images showed a homogenous distribution of chondrocytes within the ECM and displayed a spherical morphology of cells over a period of 21 days throughout the culture conditions (Figure 1A–E). Immunohistochemical staining of cartilage-characteristic collagen type II revealed the secretion of this protein as a component of the formed matrix in 3D cartilage micromasses after 14 days (starting point) and after 7 days stimulation with TNF-α or treatment with HA regardless of the combination with TNF-α (Figure 1F–J).

During culture, micromasses have developed an ECM rich in proteoglycans at day 14 (starting point), histologically detected by Safranin-O staining (Figure 1K). Afterwards micromasses were treated for further 7 days with TNF-α alone or with HA under normal and OA-like conditions, where the addition of TNF-α expedited OA pattern-oriented changes including GAG depletion. The histological sections from these samples showed less accumulation of GAGs in TNF-α-treated groups with marginal increase in HyaTNF in comparison to TNF-α (Figure 1L–O). HA alone led to a non-significant increase of GAG.

**Figure 2:**
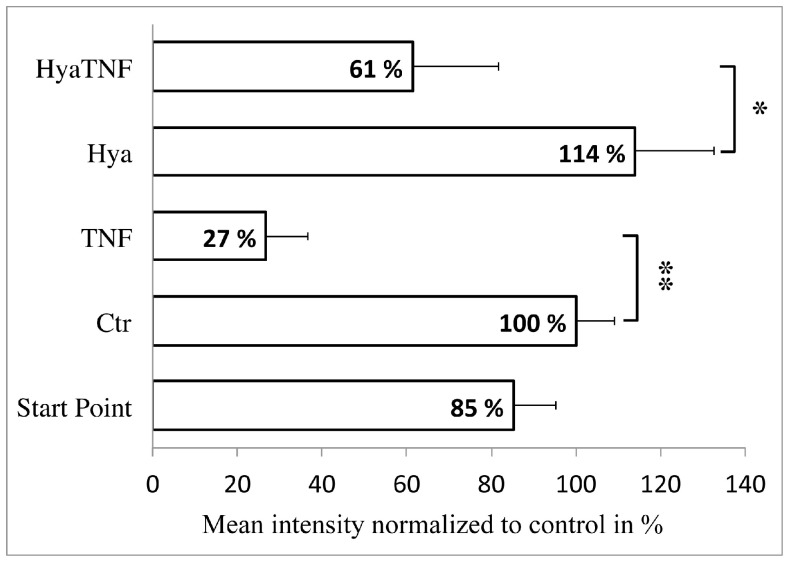
Histomorphometric analysis of Safranin-O stainings of all experimental groups considering the proportion and intensity of the stained area as the mean intensity normalized to the control + standard deviation (*n* = 3). * = *p*-value < 0.05 and ** = *p*-values < 0.01.

The histomorphometric analysis further confirmed that GAG accumulation was significantly reduced in TNF-α-treated groups compared to control groups and HA addition did not lead to any significant alterations (Figure 2). The mean intensity at starting point was 114.10 ± 10.16; after further 7 days the mean intensity of control (Ctr) was 134.86 ± 9.25. No significant differences were detected between the control group and the Hya group (Hya: 152.60 ± 18.70). TNF-α-stimulated samples had a significant decrease of GAGs regardless of HA presence (TNF: 35.81 ± 9.94, HyaTNF: 82.09 ± 20.51), although HyaTNF showed less depletion than TNF (*p*-value = 0.071). In summary, all these data demonstrated that the model we used worked properly as a highly useful approach for in vitro cartilage and OA studies. More importantly, the data showed no inhibiting or activating effect of HA on tissue-engineered normal or OA cartilage on cellular level.

### 2.2. In Vitro Model Verification by Cartilage-Related Markers

In order to gain insight into the gene expression pattern of normal and OA-like micromasses that have been treated with HA, a microarray analysis with genome-wide Affymetrix GeneChip porcine arrays was performed.

To verify the suitability of the 3D cartilage micromasses also on the molecular level, the 14-day micromasses (starting point) were examined for cartilage-related marker genes that were selected based on the data of our reference model paper (Table 1, d14_Ref) [7]. Our expression data showed the same present-absent pattern in almost all (except for *Serpin Family A Member 3, SERPINA3*) selected cartilage markers (Table 1, d14_start point) consisting of *collagen type 2, 9* (*COL2A1*, *COL9A1*), ECM connectors (*hyaluronan and proteoglycan link protein 1; HAPLN1, proline and arginine-rich end leucine-rich repeat protein; PRELP*) and other players of structural integrity of cartilage (*cartilage intermediate layer protein; CILP, cartilage oligomeric matrix protein; COMP*), enzymes (*matrix metallopeptidase 3; MMP3, serpin family A member 1; SERPINA1), growth factors (fibroblast growth factor 2; FGF2, insulin-like growth factor binding protein 3; IGFBP3*), proteoglycans (*aggrecan; ACAN, chondroitin sulphate proteoglycan 4; CSPG4*), receptors (*fibroblast growth factor receptor 1, 3; FGFR1, 3*) and transcription factors (*SRY-box 6, 9; SOX6, 9*). Further cultivation up to day 21 (Ctr) did not lead to any changes in this regard (Table 1, (d21) Ctr). In favour of demonstrating OA alterations after TNF-α stimulation, we compared the TNF-α-stimulated micromasses (TNF) with non-stimulated micromasses (Ctr). Roughly 85% of the 41 selected cartilage marker genes were significantly up- or downregulated; the fold changes (FC) are given in Table 1. These results are in line with our previously published data [7] and with the Safranin-O staining results (Figure 1K–O). In summary, these extensive similarities and the cartilage marker gene profiles given in Table 1 proved the reproducibility and suitability of the porcine micromass model for testing on the molecular level.

### 2.3. Gene Expression Profiling as Response to HA Treatment

**Figure 3:**
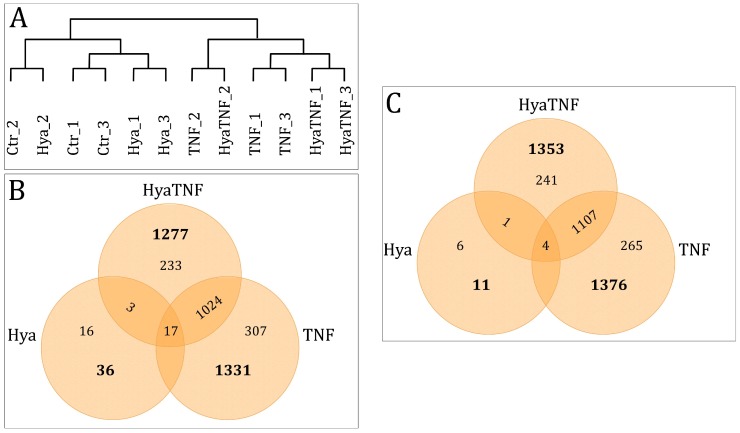
Figure 3 Gene expression profiling. (**A**) Hierarchical cluster analysis of all the probe sets. Hierarchical cluster analysis demonstrated that non-treated (Ctr) and HA-treated (Hya) tissue-engineered cartilage formed one cluster and TNF-α-treated formed another cluster, showing no significant alteration caused by HA. (**B**) Venn diagram of upregulated genes of all experimental groups (TNF, Hya and HyaTNF) compared to the control (Ctr). (**C**) Venn diagram of downregulated genes of all experimental groups (TNF, Hya and HyaTNF) compared to the control (Ctr).

In order to get a deeper insight into the HA effects, 14-day old micromasses that were further treated for 7 days with HA were compared with non-HA-treated normal and OA-like micromasses. Hierarchical clustering analysis based on all the probes of day 21 including the HA-treated groups was performed to explore the variability and similarity of gene expression at day 21 (12 samples), which therefore showed possible HA effects. Considering the expression pattern of tissue-engineered cartilage, hierarchical clustering resulted in two main groups, classified as TNF-α-treated and non-TNF-α-treated (Figure 3A). The clustering showed no distinct clustering for HA-treated samples, whereas HA-treated (without TNF-α) and normal chondrocytes (Ctr) clustered together. This similarity indicated that HA did not cause any pronounced alterations in the gene expression pattern. Therefore, no separate cluster was observed for the HA samples.

### 2.4. Overview of Differentially Expressed Genes between Controls and Treated Groups

**Figure 4:**
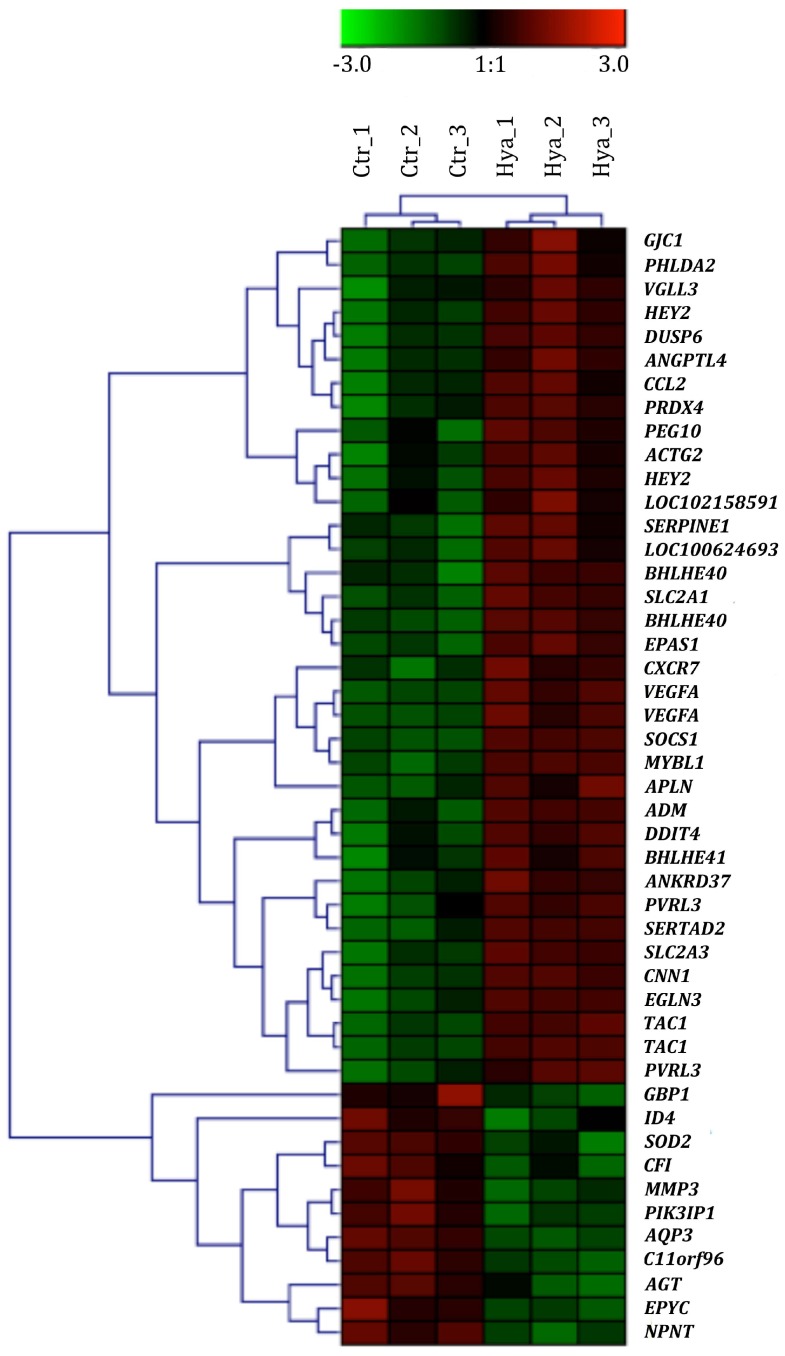
Cluster analysis based on 47 significantly differentially expressed genes. Each row depicts a single gene; each column a sample.

A Venn diagram was drawn to display the overlapping or unique members of the significantly differentially expressed genes (DEGs) between treated groups and day 21 controls. As shown in Figure 3, 16 DEGs were upregulated only in the Hya group (Figure 3B) and 6 were downregulated only in the Hya group (Figure 3C). In HyaTNF, 233 upregulated (Figure 3B) and 241 downregulated DEGs (Figure 3B) did not overlap with the other categories. The intergroup test detected only 17 upregulated (Figure 3B) and 4 downregulated DEGs (Figure 3C). A comparison between upregulated DEGs in the HyaTNF and TNF groups revealed 1024 common genes (Figure 3B) and between downregulated differentially expressed genes 1107 common genes. These results indicated similarities in the gene expression pattern of only HA-treated samples and controls, whereas TNF-α triggered different gene expression patterns regardless of HA present.

We further analysed the 47 DEGs detected between Hya and Ctr groups, including 36 upregulated and 11 downregulated genes (Figure 3A,B and Table S1). According to the heat map, the HA samples and normal micromasses (Ctr) could be well distinguished using these screened significantly regulated genes (Figure 4). The most upregulated genes (FC > 2.50) are *ankyrin repeat domain 37* (*ANKRD37*; FC = 3.00), *vascular endothelial growth factor A* (*VEGFA*; FC = 3.00), *serpin family E member 1* (*SERPINE1*; FC = 2.90), *solute carrier family 2, member 3* (*SLC2A3*; FC = 2.80) and the most downregulated genes include MMP3 (FC = −2.00), guanylate binding protein 1 (GBP1; FC = −1.80), epiphycan (EPYC; FC = −1.70) and angiotensinogen (AGT; FC= −1.70). 3 genes namely of C-C motif chemokine 2 (CCL2; FC = 2.08), vascular endothelial growth factor (VEGF; FC = 2.99) and matrix metalloproteinase 3 (MMP3; FC = −1.95) out of these 47 DEGs are involved in an arthritis pathway showing minor changes in the presence of HA in this context.

To detect the HA effect on OA-like cartilage we performed a comparative microarray analysis, which identified a total number of 101 genes that were differentially regulated between HyaTNF and TNF (Table 2). The screened DEGs were totally enriched in 35 GO terms, including 5 cellular component (CC) terms, 6 molecular function (MF) terms and 24 biological process (BP) terms according to the functional annotation. The top 20 terms are shown in Table 2, which were mainly related to CC terms such as extracellular space and basement membrane and genes enriched in these terms included *angiopoietin-like 4* (*ANGPTL4*), *apelin* (*APLN*), *C-X-C motif chemokine ligand 3* (*CXCL3*), *IGFBP3, 5, 6, COL4A1, COL14A1*, *extracellular matrix protein 1* (*ECM1*), *prostaglandin D2 synthase* (*PTGDS*), *tissue inhibitor of metalloproteinase 3* (*TIMP3*), *secreted frizzled related protein 1* (*SFRP1*) and *VEGFA*.

## 3. Discussion

The present conflicting data regarding the controversial properties of HA, the standard viscosupplement for OA [8], encouraged us to investigate more thoroughly its physiologic effect on cellular and molecular level. Sun et al. have already proved that HA provide pain relief and functional improvement in patients with ankle OA [9]. In another hypothesis-driven study it was shown that HA exhibits a pronounced suppressive effect on MMP13 [10]. Although HA effects have been studied before in patients to some extent and even during in vitro cartilage formation [11] this study has been the first, to perform a global gene expression analysis on HA-treated normal and OA-like tissue-engineered cartilage. Our findings showed no inhibiting or activating effect of HA on tissue-engineered normal or OA-like cartilage on the cellular level. On the molecular lever, we could observe minor changes in arthritis context but no pronounced alterations were caused by HA. We could also confirm that the OA model we used was a highly useful approach for in vitro cartilage and OA studies.

Since the OA chondrocytes can only maintain the pathologic condition for a short time under cell culture conditions, long-term evaluations require the addition of OA-inducing agents. TNF-α and IL1 are the most widely used cytokines to induce OA as well as rheumatoid arthritis. To induce OA changes the inflammatory mediator TNF-α was used in this study. TNF-α is increased in the synovial fluid of OA patients and is known to mediate the catabolic process [12]. The TNF-α concentration in the synovial fluid of OA patients is in the lower range (e.g., 0.124 ± 1.59 pg/mL) [13] but in vitro the concentration should be much higher (10 ng/mL) to achieve alterations in a shorter time.

Live-dead staining of 14-day micromasses, which were further treated for 7 days with 0.3 wt % HA in normal and OA-like conditions (where TNF-α was added to simulate important aspects of OA), revealed a majority of viable cells embedded in ECM. A normal morphology and even distribution of cells through the matrix was observed in all experimental groups. On gene expression level, TNF-α addition led to an induction of genes related to cell death such as *TNFSF10*, *PMAIP1*, *AHR* and *ADM* that was not altered by the addition of HA. Consequently, HA did not prevent the induction of pathways related to cell death such as apoptosis and autophagy but HA did not cause any cell death stimulation under normal and OA-like conditions compared to control group and starting point. We used 0.3 wt % concentration of HA to mimic the in vivo situation, because in healthy human synovial fluid, a broad range of HA concentrations was measured ranging between 0.05 and 0.4 wt %, with 0.3 wt % being typical [14]. Collagen type II immunostaining did not reveal any specific differences between experimental groups, which is in line with the previous published data [7]. It has been shown that the total collagen loss was not pronounced in this model and HA did not seem to change this pattern either. Smyth et al. have recently shown in a rabbit model that addition of HA caused no noticeable difference in the type-II collagen immunoreaction between the HA-treated grafts and the controls [15]. Proteoglycan depletion plays a main role in the histopathological assessment of OA grade [16] and is a detectable feature in this model. Addition of TNF-α, a well-known mediator of acute inflammation in cartilage pathology, triggered a clear depletion of GAG in HA-treated as well as non-treated micromasses. However, there was less decrease of GAG observed in HyaTNF. This can be explained by the study of Greenberg et al. who concluded from their cartilage synovium co-culture model that HA inhibits the MMP- and IL1-mediated decrease in glycosaminoglycan production by cartilage explants [17] and this chondrostimulative effect was further confirmed by Elmorsy et al. in vivo [4]. These observations in connection with gene expression alterations caused by addition of TNF-α (Table 1) showed the feasibility of the porcine micromass model to assess HA influence on normal and OA cartilage.

Comparative genome-wide expression analysis of porcine micromasses treated with HA and the non-treated micromasses revealed a total of 47 dysregulated genes (Figure 3) including up-regulation of *ANKRD37, VEGFA, SERPINE1, SLC2A3* as well as gene coding for chemokine *CCL2* and downregulation of *MMP3, GBP1, EPYC* and *AGT*. ANKRD37 is associated with hypoxia and cell respond to hypoxic environment is upregulation of *ANKRD37* RNA. Here the HA provoked the same response. It has been shown that this could lead to increased cartilage-specific gene expression, for example, *ACAN* and *Sox9* [18]. This could be the reason why we observed an insignificant increase in GAG content of HyaTNF. VEGFA has a role in cartilage maturation and is critical for chondrocyte survival [19]. Its upregulation in this study together with *ANKRD37* upregulation confirms the existing findings that HA can reduce chondrocyte apoptosis and enhance ECM synthesis [20]. SERPINE1 has a function in complement cascade and its upregulation has been reported in OA-affected cartilage [21]. *SLC2A3* encodes GLUT3, a glucose transporter and plays an essential role in chondrocyte metabolism and physiology and can also be upregulated as a result of hypoxia. This hypoxia-like influence of HA can be explained due to its high viscosity that restrains the diffusion of oxygen. Previous studies have demonstrated that HA has the potential to inhibit the activity of matrix metalloproteinases and catabolic cytokines [22]. In the present study, we did not detect any significant changes in MMPs by the addition of HA in the OA-like condition. Interestingly, the *TIMP3* was upregulated in HA-treated OA-like micromasses. The chondroprotective role of TIMP3 is illustrated by studies showing that mice lacking the gene for TIMP3 develop accelerated OA as they age [23] and, conversely, that recombinant TIMP3 inhibits development of OA in a rat model of disease [24]. Although no HA-dependent alterations in collagen type II deposition were detected, the finding is in line with previous studies. GBP1 is an enzyme-binding protein, which showed an increase under rheumatoid arthritic conditions [25] and HA-treated micromasses showed the reverse trend. We have also observed the downregulation of *EPYC,* which is a marker enriched in growth plate cartilage and is used to identify hyaline cartilage subtype [26].

We have further compared gene expression of HyaTNF group to TNF in order to study the genes that are dysregulated by HA treatment under OA conditions. We found an increased level of *IGF-binding proteins* (*IGFBP*) in HyaTNF. *Insulin-like growth factor 1* (*IGF1*) is the most likely candidate to affect the anabolism (synthesis of both collagen type II and proteoglycan core protein) of cartilage matrix molecules and IGFBPs have a high affinity for IGF1 [27]. From our data (Table 2) we can conclude that HA can possibly influence the cartilage anabolism via binding to IGFs and stabilize the chondrocyte phenotype in pathological conditions. CXCL3 chemokine has been reported to have an increased expression in OA cartilage [28]. HA seems to hamper this event by 2-fold downregulation. GPX3 is involved in oxidative damage defence and is downregulated in OA cartilage [29]. In our study HA appears to amplify this trend (FC = −2.6) in OA-like cartilage but not in healthy micromasses, which shows that the effect is caused by TNF-α addition and not by HA. 

HA with different molecular weight and consistencies are known to have different clinical outcomes. In our study, we used one type of HA, namely OSTENIL^®^. Therefore, further investigation of more HAs with different molecular weights and concentrations is necessary.

Based on our previous study, a sulphated polyethylene glycol hydrogel with anti-inflammatory properties has viscoelastic properties that are comparable to HA for intra-articular injection, where for medical applications the above-mentioned hydrogel has the advantage of being much less easily displaced from its injection place than HA [30]. Comparing HA with such alternative candidates that have disease-modifying properties is required for the development of better therapeutics.

This study has several limitations, including lack of verification for the findings on protein level, which can lead to an increased evidence level of the study. Furthermore, the pathophysiological changes in OA do not only influence articular cartilage but also other parts of the synovial joint such as subchondral bone and joint capsule. Therefore, the effect of HA needs to be further investigated thoroughly on these tissues as well. Another limitation is that the HA concentration used in this study is thinner than the actual clinical concentration to enable the pipetting and the exchange of metabolites.

## 4. Materials and Methods

### 4.1. Chondrocyte Isolation

Chondrocytes were isolated from the medial and lateral femoral condyle cartilage of 9 domestic pigs according to a previously published protocol [31]. No animal approval was needed because the samples were obtained from a local slaughterhouse. Briefly, cartilage pieces were incubated for 19 h in spinner flasks containing Roswell Park Memorial Institute (RPMI) medium, supplemented with 10% foetal bovine serum (FBS, Thermo Fisher Scientific, Dreieich, Germany), 100 U/mL penicillin (Pen) and 100 µg/mL streptomycin (Strep), 333.3 U/mL collagenase II (all Merck, Darmstadt, Germany), 1 U/mL collagenase P (Roche Diagnostics, Mannheim, Germany) and 33.3 U/mL hyaluronidase (Sigma-Aldrich, Steinheim, Germany). Afterwards, cell suspensions were filtered through a 100 µm nylon mesh (Becton Dickinson, Heidelberg, Germany), washed in Hanks solution (Merck) and resuspended in culture medium consisting of RPMI, 10% FBS, 100 U/mL Pen, 100 µg/mL Strep and 170 µM l-ascorbic acid (Sigma-Aldrich).

### 4.2. Preparation of 3D-Chondrocyte Micromass Cultures

After chondrocyte isolation from 9 donors, cells of three different donors were pooled together in one sample pool, resulting in three different sample pools. A volume of 200 µL containing 6 × 10^5^ freshly isolated chondrocytes in culture medium was transferred to each well of 96-well flat bottom plates (Becton Dickinson) to generate a high-density micromass culture (tissue-engineered cartilage) [7]. Subsequently, the culture plates were incubated for 24 h (37 °C, 5% CO_2_) to ensure cell sedimentation. The medium was changed daily. Micromasses were allowed to form ECM for 14 days and then were treated for further 7 days with HA alone (MW=1.2 KDa; OSTENIL^®^, TRB Chemedica, Germany), TNF-α alone (R&D Systems, Wiesbaden, Germany) to induce OA-like changes, or in combination thereof. This resulted in 5 experimental groups: (1) micromasses cultured for 14 days (start point) and (2) further cultured for 7 days without treatment (control; Ctr) or treatment (3) with 0.3 wt % HA diluted in culture medium (Hya), (4) 0.6 nmol/l TNF-α diluted in culture medium, or (5) a combination of (3) and (4) (HyaTNF).

### 4.3. Live/Dead Assay

To demonstrate the cell viability of the micromasses, propidium iodide/fluorescein diacetate (PI/FDA) staining (Sigma-Aldrich) was performed. The micromasses were washed with PBS and stained with FDA under darkness. To prepare the FDA staining solution 1 mg/mL FDA were dissolved in acetone and further diluted to a concentration of 3 µg/mL in PBS. Then, the samples were rinsed with PBS before being counterstained with PI. To prepare the PI staining, 1 mg/mL PI were dissolved in distilled water and further diluted to a concentration of 0.1 mg/mL in PBS. After an additional washing step, the micromasses were analysed under a fluorescent microscope (Olympus CKX41, Hamburg, Germany).

### 4.4. Histological and Immunohistochemical Staining

To document ECM formation or loss, micromasses that were embedded in an optimal cutting temperature compound (Sakura Finetek, Staufen im Breisgau, Germany) were cryosectioned at 8 µm and mounted on glass slides. Sulphated cartilage glycosaminoglycans (GAGs) were stained with 0.7% Safranin-O in 67% ethanolic solution and cell nuclei were counterstained with 0.2% Fast Green in 0.3% acetic acid. Stainings were photodocumented using a light microscope (AX 10, Zeiss, Jena, Germany).

The intensity of the Safranin-O staining is directly proportional to the GAG amount of the tissue and can therefore be called a semi-quantitative histochemical method [32]. Therefore a histomorphometric analysis was performed as previously described [7]. Briefly, pictures were taken and all pixels in the areas of interest were valued in the RGB colour mode with a tool based on Xcode (Apple, Sunnyvale, CA, USA). When the red value (R) multiplied by 2 was higher than the sum of the green (G) and blue (B) values, the pixel was counted as red. The intensity of each red pixel was calculated with this formula: intensity = 2 × *R*-value − *G*-value − *B*-value. Values of the intensity ranged between 1 and 510. The mean intensity (sum of intensities/area of interest) was calculated from each image.

Collagen type II expression was analysed by immunohistochemistry with polyclonal mouse anti-porcine type II collagen antibodies (Calbiochem CP18, Merck, Darmstadt, Germany). Mouse IgG (DAKO, Hamburg, Germany) served as a control. EnVision detection antibody was used to visualize collagen type II antibodies and nuclei were counterstained with hematoxylin (DAKO).

### 4.5. RNA Isolation

Total RNA was isolated from micromasses that were cultured over 21 days. For each individual replicate (*n* = 3) of each experimental group, 5 micromasses were snap-frozen in liquid nitrogen and stored at −80 °C until further use. The frozen samples were transferred to 1 mL TriReagent (Sigma-Aldrich) and mechanically homogenized. Subsequently, 133 μL 1-bromo-3-chloro-propane (Sigma-Aldrich) was admixed followed by centrifugation for 45 min at 13,000× *g*. The aqueous phase was collected and supplemented with same volume of 70% ethanol. Further purification was performed according to protocol for animal tissues of the RNeasy Mini Kit (Qiagen, Hilden, Germany). The RNA concentration was determined by the Nanodrop 1000 spectrophotometer (Thermo Fisher Scientific). The integrity of the RNA was determined by the Agilent Bioanalyzer 2100 (Agilent Technologies, Santa Clara, CA, USA). The RNA samples used in this study had an integrity number above 8.9.

### 4.6. Microarray Analysis

Altogether, data from 15 microarray experiments (5 groups in triplicates) are included in this study, from which selected data of 6 microarrays (triplicates of Ctr and TNF groups) have already been published in a study with a totally different focus on sulphated polyethyleneglycol hydrogels as a possible HA alternative [33].

A total of 23,937 probe sets representing 20,201 porcine genes were covered in the Affymetrix GeneChip porcine array (Affymetrix, Freiburg, Germany). RNA processing and hybridization were performed according to the manufacturer’s protocol. The GeneChips were scanned with the Affymetrix GeneChip scanner 3000. Raw gene expression data were normalized and analysed with GeneChip operating software 1.4 (GCOS, Affymetrix). Comparisons between triplicates of the starting point group and the Ctr group were performed on the basis of a cartilage-marker list associated with our in vitro OA model [7]. Other paired group comparisons were performed between replicates of each group (9 comparisons). Genes were selected for further analysis that showed (1) a significant signal change, which was detected by GCOS for at least 7 out of 9 comparisons, (2) a 1.5 mean-fold change and (3) a *p*-value < 0.05 applying *t*-test. To group genes with coherent expression profiles into modules, we used complete linkage hierarchical clustering (HCL) with normalized log 2-transformed signals. A Pearson correlation was done to determine the distance measure and complete linkage clustering by agglomeration rule, using Genesis 1.7.6 software [34]. Gene ontology (GO) terms analysis, biological process (BP) function enrichment analysis and Kyoto encyclopaedia of genes and genomes (KEGG) pathway enrichment analysis of differentially expressed genes was performed using the database for annotation, visualization and integrated discovery (DAVID) [35]. In order to find the names for unnamed porcine probe set IDs, we used cross-species relationships between porcine and human probe set IDs (U133PlusVsPorcine_Complex sheet) in combination with human NetAffx annotation file (HG_U133_Plus_2 Array, Affymetrix).

### 4.7. Statistical Analysis

The intensity of the Safranin-O stained areas are shown as the mean intensity normalized to the control and standard deviation. A *p*-value lower than 0.05 was accepted as statistically significant. The significance level of log 2-tranformed microarray data was determined with the independent two sample t-test statistics of the Excel 2011 software package (Microsoft, Redmond, WA, USA). The normality of distribution was investigated applying the Anderson-Darling test [36] and the equal variance of the compared sample groups was tested applying the f test [37,38]. The data showed normal distribution with equal variance, therefore t-test was applied. Differences were considered significant at *p* < 0.05.

The raw data is available for presentation to the referees and the editors of the journal, if requested. The microarray data will be deposited in the GEO database.

## 5. Conclusions

In conclusion, the present study can further confirm that HA has a minor physiological impact on normal and OA-like tissue-engineered cartilage compared to alterations caused by TNF-α. Nevertheless, it can possibly influence the cartilage anabolism via stabilizing the chondrocyte phenotype in pathological conditions. Moreover, the upregulation of *VEGFA* and *ANKRD37* genes confirms the chondrostimulative potential of HA and slow down degradation. Understanding these HA-related modifications may serve as a guide toward imminent therapies. In addition to providing a more comprehensive picture of HA influence, the results in this study further validate the feasibility of in vitro OA model for the investigation of HA.

## Figures and Tables

**Table 1:** Overview of gene expression of selected cartilage markers.

	Gene Symbol	Gene Name	d14_Ref *	(d14) Start Point	(d21) Ctr	FC Trend
Collagens	*COL1A2*	collagen type I α2	**+**	**+**	**+**	−2.7
	*COL2A1*	collagen type II α1	**+**	**+**	**+**	−25.8
	*COL9A2*	collagen type IX α1	**+**	**+**	**+**	−11.0
ECM connectors	*FMOD*	fibromodulin	**+**	**+**	**+**	−1.3
	*HAPLN1*	hyaluronan and proteoglycan link protein 1	**+**	**+**	**+**	−9.5
	*LGALS3*	carbohydrate-binding protein 35	**+**	**+**	**+**	1.2
	*PCOLCE2*	C-endopeptidase enhancer 2	**+**	**+**	**+**	/
	*PRELP*	prolargin	**+**	**+**	**+**	−1.9
Enzymes	*MMP3*	matrix metallopeptidase 3	**+**	**+**	**+**	19.9
	*SERPINA1*	serpin peptidase inhibitor clade A member 1	**+**	**+**	**+**	−31.3
	*SERPINA3*	serpin peptidase inhibitor clade A member 3	-	+	**+**	/
Growth factors	*BMP2*	bone morphogenetic protein 2	+	**+**	**+**	/
	*BMP4*	bone morphogenetic protein 4	**+**	**+**	**+**	−1.8
	*BMP7*	bone morphogenetic protein 7	-	-	-	3.2
	*FGF18*	fibroblast growth factors 18	-	-	-	−2.6
	*FGF2*	fibroblast growth factors 2	**+**	**+**	**+**	4.6
	*FGF9*	fibroblast growth factors 9	-	-	-	−3.6
	*IGF1*	insulin-like growth factor 1	**+**	**+**	**+**	−2.8
	*IGFBP3*	insulin-like growth factor binding protein 3	**+**	**+**	**+**	4.2
	*TGFB1*	transforming growth factor beta 1	**+**	**+**	**+**	−1.6
	*TGFB2*	transforming growth factor beta 2	**+**	**+**	**+**	1.6
	*TGFB3*	transforming growth factor beta 3	**+**	**+**	**+**	−2.6
	*THRA*	thyroid hormone receptor α	**+**	**+**	**+**	−1.7
Proteoglycan	*ACAN*	aggrecan	**+**	**+**	**+**	−13.1
	*BGN*	biglycan	**+**	**+**	**+**	−2.6
	*CSPG4*	chondroitin sulphate proteoglycan 4	**+**	**+**	**+**	−3.8
	*DCN*	decorin	**+**	**+**	**+**	−2.4
	*HSPG2*	heparan sulphate proteoglycan 2	**+**	**+**	**+**	/
	*VCAN*	versican	**+**	**+**	**+**	/
Receptors	*FGFR1*	fibroblast growth factor receptor 1	**+**	**+**	**+**	−1.2
	*FGFR2*	fibroblast growth factor receptor 2	**+**	**+**	**+**	−1.7
	*FGFR3*	fibroblast growth factor receptor 3	**+**	**+**	**+**	−2.5
Structural integrity of cartilage	*CHI3L1*	cartilage intermediate layer protein	**+**	**+**	**+**	1.8
	*CILP*	cartilage oligomeric protein	**+**	**+**	**+**	7.9
	*COMP*	extracellular matrix protein 1	**+**	**+**	**+**	−8.6
	*ECM1*	fibrillin 1	**+**	**+**	**+**	4.0
	*FBN1*	fibronectin 1	**+**	**+**	**+**	2.4
	*FN1*	matrix gla protein	**+**	**+**	**+**	/
	*MGP*	cartilage intermediate layer protein	**+**	**+**	**+**	−2.1
Transcription factors	*SOX6*	SRY (Sex Determining Region Y)-Box 6	**+**	**+**	**+**	−3.3
	*SOX9*	SRY (Sex Determining Region Y)-Box 9	**+**	**+**	**+**	/

**+** (Bold) = present in all 3 replicates, + = present in some replicates, - = absent in all replicates, / = no significant fold change, FC = fold change. * = [7].

**Table 2:** The top 20 GO terms from enrichment analysis of DEGs (HyaTNF vs. TNF) sorted according to *p*-value.

Category	GO ID	GO Name	Gene Nr.	*p*-Value	Genes
CC	GO:0005615	extracellular space	16	3.0 × 10^−4^	*CXCL3, IGFBP6, LMCD1, PLBD1, ECM1, TIMP3, COL14A1, PTGDS, SFRP1, HIST2H2BE, GPX3, VEGFA, IGFBP3, APLN, ANGPTL4, IGFBP5*
MF	GO:0001968	fibronectin binding	3	2.3 × 10^−4^	*VEGFA, IGFBP3, IGFBP5*
MF	GO:0031994	insulin-like growth factor I binding	3	3.4 × 10^−4^	*IGFBP6, IGFBP3, IGFBP5*
MF	GO:0031995	insulin-like growth factor II binding	3	3.4 × 10^−4^	*IGFBP6, IGFBP3, IGFBP5*
BP	GO:0043567	regulation of insulin-like growth factor receptor signalling pathway	3	6.9 × 10^−4^	*IGFBP6, IGFBP3, IGFBP5*
CC	GO:0005604	basement membrane	4	1.8 × 10^−3^	*P3H2, COL4A1, ITGA6, TIMP3*
BP	GO:0071456	cellular response to hypoxia	4	2.1 × 10^−3^	*PTGS2, SFRP1, VEGFA, ANGPTL4*
BP	GO:0045663	positive regulation of myoblast differentiation	3	3.7 × 10^−3^	*CDON, BOC, IGFBP3*
BP	GO:0045892	negative regulation of transcription, DNA-templated	6	9.5 × 10^−3^	*CRY2, SFRP1, BEND5, CCDC85B, BASP1, HMGA1*
CC	GO:0005576	extracellular region	8	9.7 × 10^−3^	*FGF7, PTGDS, PAPPA, AGT, NMB, FGF13, CFD, GHR*
BP	GO:0001558	regulation of cell growth	3	1.4 × 10^−2^	*IGFBP6, IGFBP3, IGFBP5*
BP	GO:0017148	negative regulation of translation	3	1.7 × 10^−2^	*BTG2, ENC1, IGFBP5*
CC	GO:0042567	insulin-like growth factor ternary complex	2	1.9 × 10^−2^	*IGFBP3, IGFBP5*
CC	GO:0070062	extracellular exosome	18	2.2 × 10^−2^	*SCPEP1, IGFBP6, NPR3, ECM1, TIMP3, ARG1, COL14A1, BTG2, SFRP1, PTGDS, RAB19, HIST2H2BE, AGT, PCBP2, BLVRB, GPX3, IGFBP3, MEST*
BP	GO:0044342	type B pancreatic cell proliferation	2	3.5 × 10^-2^	*IGFBP3, IGFBP5*
BP	GO:0014912	negative regulation of smooth muscle cell migration	2	3.5 × 10^−2^	*IGFBP3, IGFBP5*
BP	GO:0006979	response to oxidative stress	3	3.8 × 10^−2^	*PTGS2, GPX3, SRXN1*
BP	GO:0043568	positive regulation of insulin-like growth factor receptor signalling pathway	2	4.0 × 10^−2^	*IGFBP3, IGFBP5*
BP	GO:0045893	positive regulation of transcription, DNA-templated	5	4.0 × 10^−2^	*FGF7, SFRP1, AGT, SERTAD3, HMGA1*

## Supplementary Materials

Supplementary materials can be found at http://www.mdpi.com/1422-0067/19/5/1519/s1. Table S1: Signal values of 47 differentially expressed genes and SLR (signal log ratios) between Ctr and Hya.

## Abbreviations

ACAN	aggrecan
AGT	angiotensinogen
ANGPTL4	included angiopoietin-like 4
ANKRD37	ankyrin repeat domain 37
APLN	apelin
BP	biological process
CC	cellular component
CCL2	C-C motif chemokine 2
CD44	cluster of differentiation 44
CILP	cartilage intermediate layer protein
COL2A1	collagen type 2 alpha 1 chain
COL9A1	collagen type IX alpha 1 chain
COMP	cartilage oligomeric compound
CSPG4	chondroitin sulphate proteoglycan 4
CXCL3	C-X-C motif chemokine ligand 3
DAVID	database for annotation, visualization and integrated discovery
ECM	extra cellular matrix
EPYC	epiphycan
FBS	foetal bovine serum
FC	fold change
FGF	fibroblast growth factor
FGFR	fibroblast growth factor receptor
GAG	glycosaminoglycan
GBP1	guanylate binding protein 1
GCOS	GeneChip operating software
GO	gene ontology
HA	hyaluronic acid
HAPLN1	hyaluronan and proteoglycan link protein 1
HCL	hierarchical clustering
IGFBP	insulin-like growth factor binding protein
IL	interleukin
KEGG	Kyoto encyclopaedia of genes and genomes
MF	molecular function
MMP	matrix metalloproteinase
OA	osteoarthritis
Pen	penicillin
PI/FDA	propidium iodide/fluorescein diacetate
PRELP	proline and arginine-rich end leucine-rich repeat protein
PTGDS	prostaglandin D2 synthase
SERPINA	serpin family A member
SFRP1	secreted frizzled related protein 1
SLC2A3	solute carrier family 2 member 3
SOX	SRY-Box
Strep	streptomycin
TIMP3	TIMP metallopeptidase inhibitor 3
TNF-α	tumour-necrosis factor-alpha
VEGFA	vascular endothelial growth factor A

## References

[1] Karna E., Miltyk W., Surażyński A., Pałka J.A. (2008). Protective effect of hyaluronic acid on interleukin-1-induced deregulation of β1-integrin and insulin-like growth factor-I receptor signalling and collagen biosynthesis in cultured human chondrocytes. Mol. Cell. Biochem..

[2] Swann D., Radin E., Nazimiec M., Weisser P., Curran N., Lewinnek G. (1974). Role of hyaluronic acid in joint lubrication. Ann. Rheum. Dis..

[3] Kosinska M.K., Ludwig T.E., Liebisch G., Zhang R., Siebert H.C., Wilhelm J., Kaesser U., Dettmeyer R.B., Klein H., Ishaque B. (2015). Articular Joint Lubricants during Osteoarthritis and Rheumatoid Arthritis Display Altered Levels and Molecular Species. PLoS ONE.

[4] Elmorsy S., Funakoshi T., Sasazawa F., Todoh M., Tadano S., Iwasaki N. (2014). Chondroprotective effects of high-molecular-weight cross-linked hyaluronic acid in a rabbit knee osteoarthritis model. Osteoarthr. Cartil..

[5] Moreland L.W. (2003). Intra-articular hyaluronan (hyaluronic acid) and hylans for the treatment of osteoarthritis: Mechanisms of action. Arthritis Res. Ther..

[6] Hunter C.J., Levenston M.E. (2004). Maturation and integration of tissue-engineered cartilages within an in vitro defect repair model. Tissue Eng..

[7] Schlichting N., Dehne T., Mans K., Endres M., Stuhlmüller B., Sittinger M., Kaps C., Ringe J. (2014). Suitability of porcine chondrocyte micromass culture to model osteoarthritis in vitro. Mol. Pharm..

[8] Haward S.J., Jaishankar A., Oliveira M., Alves M., McKinley G. (2013). Extensional flow of hyaluronic acid solutions in an optimized microfluidic cross-slot device. Biomicrofluidics.

[9] Sun S.-F., Chou Y.-J., Hsu C.-W., Hwang C.-W., Hsu P.-T., Wang J.-L., Hsu Y.-W., Chou M.-C. (2006). Efficacy of intra-articular hyaluronic acid in patients with osteoarthritis of the ankle: A prospective study. Osteoarthr. Cartil..

[10] Pohlig F., Guell F., Lenze U., Lenze F.W., Muhlhofer H.M., Schauwecker J., Toepfer A., Mayer-Kuckuk P., von Eisenhart-Rothe R., Burgkart R. (2016). Hyaluronic Acid Suppresses the Expression of Metalloproteinases in Osteoarthritic Cartilage Stimulated Simultaneously by Interleukin 1β and Mechanical Load. PLoS ONE.

[11] Responte D.J., Natoli R.M., Athanasiou K.A. (2012). Identification of potential biophysical and molecular signalling mechanisms underlying hyaluronic acid enhancement of cartilage formation. J. R. Soc. Interface.

[12] Smith M.D., Triantafillou S., Parker A., Youssef P., Coleman M. (1997). Synovial membrane inflammation and cytokine production in patients with early osteoarthritis. J. Rheumatol..

[13] Ozler K., Aktas E., Atay C., Yilmaz B., Arikan M., Gungor S. (2016). Serum and knee synovial fluid matrix metalloproteinase-13 and tumour necrosis factor-alpha levels in patients with late-stage osteoarthritis. Acta Orthop. Traumatol. Turc..

[14] Balazs E.A., Watson D., Duff I.F., Roseman S. (1967). Hyaluronic acid in synovial fluid. I. Molecular parameters of hyaluronic acid in normal and arthritic human fluids. Arthritis Rheumatol..

[15] Smyth N.A., Ross K.A., Haleem A.M., Hannon C.P., Murawski C.D., Do H.T., Kennedy J.G. (2017). Platelet-Rich Plasma and Hyaluronic Acid Are Not Synergistic When Used as Biological Adjuncts with Autologous Osteochondral Transplantation. Cartilage.

[16] Pritzker K., Gay S., Jimenez S., Ostergaard K., Pelletier J.-P., Revell P., Salter D., van den Berg W. (2006). Osteoarthritis cartilage histopathology: Grading and staging. Osteoarthr. Cartil..

[17] Greenberg D., Stoker A., Kane S., Cockrell M., Cook J. (2006). Biochemical effects of two different hyaluronic acid products in a co-culture model of osteoarthritis. Osteoarthr. Cartil..

[18] Foldager C.B., Nielsen A.B., Munir S., Ulrich-Vinther M., Søballe K., Bünger C., Lind M. (2011). Combined 3D and hypoxic culture improves cartilage-specific gene expression in human chondrocytes. Acta Orthop..

[19] Zelzer E., Mamluk R., Ferrara N., Johnson R.S., Schipani E., Olsen B.R. (2004). VEGFA is necessary for chondrocyte survival during bone development. Development.

[20] Altman R., Manjoo A., Fierlinger A., Niazi F., Nicholls M. (2015). The mechanism of action for hyaluronic acid treatment in the osteoarthritic knee: A systematic review. BMC Musculoskelet. Disord..

[21] Ramos Y.F., den Hollander W., Bovee J.V., Bomer N., van der Breggen R., Lakenberg N., Keurentjes J.C., Goeman J.J., Slagboom P.E., Nelissen R.G. (2014). Genes involved in the osteoarthritis process identified through genome wide expression analysis in articular cartilage; the RAAK study. PLoS ONE.

[22] Julovi S.M., Ito H., Nishitani K., Jackson C.J., Nakamura T. (2011). Hyaluronan inhibits matrix metalloproteinase-13 in human arthritic chondrocytes via CD44 and P38. J. Orthop. Res..

[23] Sahebjam S., Khokha R., Mort J.S. (2007). Increased collagen and aggrecan degradation with age in the joints of TIMP3^−/−^ mice. Arthritis Rheumatol..

[24] Black R., Castner B., Slack J., Tocker J., Eisenman J., Jacobson E., Delaney J., Winters D., Hecht R., Bendele A. (2006). A14 Injected TIMP-3 Protects Cartilage in A Rat Meniscal Tear Model. Osteoarthr. Cartil..

[25] Andreas K., Lübke C., Häupl T., Dehne T., Morawietz L., Ringe J., Kaps C., Sittinger M. (2008). Key regulatory molecules of cartilage destruction in rheumatoid arthritis: An in vitro study. Arthritis Res. Ther..

[26] Leijten J.C., Emons J., Sticht C., van Gool S., Decker E., Uitterlinden A., Rappold G., Hofman A., Rivadeneira F., Scherjon S. (2012). Gremlin 1, frizzled-related protein and Dkk-1 are key regulators of human articular cartilage homeostasis. Arthritis Rheumatol..

[27] Martel-Pelletier J., di Battista J., Lajeunesse D., Pelletier J.-P. (1998). IGF/IGFBP axis in cartilage and bone in osteoarthritis pathogenesis. Inflamm. Res..

[28] Karlsson C., Dehne T., Lindahl A., Brittberg M., Pruss A., Sittinger M., Ringe J. (2010). Genome-wide expression profiling reveals new candidate genes associated with osteoarthritis. Osteoarthr. Cartil..

[29] Aigner T., Fundel K., Saas J., Gebhard P.M., Haag J., Weiss T., Zien A., Obermayr F., Zimmer R., Bartnik E. (2006). Large-scale gene expression profiling reveals major pathogenetic pathways of cartilage degeneration in osteoarthritis. Arthritis Rheumatol..

[30] Von Lospichl B., Hemmati-Sadeghi S., Dey P., Dehne T., Haag R., Sittinger M., Ringe J., Gradzielski M. (2017). Injectable hydrogels for treatment of osteoarthritis–A rheological study. Colloids Surf. B.

[31] Lübke C., Ringe J., Krenn V., Fernahl G., Pelz S., Kreusch-Brinker R., Sittinger M., Paulitschke M. (2005). Growth characterization of neo porcine cartilage pellets and their use in an interactive culture model. Osteoarthr. Cartil..

[32] Rosenberg L. (1971). Chemical basis for the histological use of safranin O in the study of articular cartilage. J. Bone Jt. Surg..

[33] Hemmati-Sadeghi S., Dey P., Ringe J., Haag R., Sittinger M., Dehne T. (2018). Biomimetic sulphated polyethylene glycol hydrogel inhibits proteoglycan loss and tumour necrosis factor-α-induced expression pattern in an osteoarthritis in vitro model. J. Biomed. Mater. Res. Part B.

[34] Sturn A., Quackenbush J., Trajanoski Z. (2002). Genesis: Cluster analysis of microarray data. Bioinformatics.

[35] Dennis G., Sherman B.T., Hosack D.A., Yang J., Gao W., Lane H.C., Lempicki R.A. (2003). DAVID: Database for annotation, visualization and integrated discovery. Genome Biol..

[36] Anderson T.W., Darling D.A. (1952). Asymptotic theory of certain “goodness of fit” criteria based on stochastic processes. Ann. Math. Stat..

[37] Box G.E. (1953). Non-normality and tests on variances. Biometrika.

[38] Ahmed E.M. (2015). Hydrogel: Preparation, characterization and applications: A review. J. Adv. Res..

